# Supplementary material for: Visualizing ’omic feature rankings and log-ratios using Qurro
Source: NAR Genom Bioinform. 2020 Apr 28;2(2):lqaa023. doi: 10.1093/nargab/lqaa023 (PMC7194218; doi:10.1093/nargab/lqaa023)
Supplement: lqaa023_Supplemental_File [file lqaa023_supplemental_file.pdf]

Published online DD MONTH YYYY *Nucleic Acids Research, 2019, Vol. VOLNUM, No. ISSNUM* **1–3**
doi:10.1093/nar/gkn000

Visualizing 'omic feature rankings and log-ratios using Qurro: Supplementary Information

Marcus W. Fedarko [1,2], Cameron Martino [2,3], James T. Morton [4], Antonio González [5], Gibraan Rahman [3], Clarisse A. Marotz [6], Jeremiah J. Minich [7], Eric E. Allen [2,7,8], and Rob Knight [1,2,5,9,*]

[1]Department of Computer Science and Engineering, University of California San Diego, 9500 Gilman Drive, La Jolla, CA, 92093, USA, [2]Center for Microbiome Innovation, University of California San Diego, 9500 Gilman Drive, La Jolla, CA, 92093, USA, [3]Bioinformatics and Systems Biology Program, University of California San Diego, 9500 Gilman Drive, La Jolla, CA, 92093, USA, [4]Flatiron Institute, Simons Foundation, 162 Fifth Avenue, New York City, NY, 10010, USA, [5]Department of Pediatrics, University of California San Diego, 9500 Gilman Drive, La Jolla, CA, 92093, USA [6]Department of Biomedical Sciences, University of California San Diego, 9500 Gilman Drive, La Jolla, CA, 92093, USA [7]Marine Biology Research Division, Scripps Institution of Oceanography, University of California San Diego, 9500 Gilman Drive, La Jolla, CA, 92093, USA, [8]Department of Biological Sciences, University of California San Diego, 9500 Gilman Drive, La Jolla, CA, 92093, USA, [9]Department of Bioengineering, University of California San Diego, 9500 Gilman Drive, La Jolla, CA, 92093, USA

1. COMPUTING FEATURE DIFFERENTIALS USING SONGBIRD

As discussed in the main text, the initial focus of this re-analysis was on visualizing the associations of features with different *Scomber japonicus* body sites. To assess this, we ran Songbird (Morton *et al.*, 2019) using the formula `C(sample_type_body_site, Treatment('sea water'))`. This produced six fields of differentials:

1. `Intercept`
2. `C(sample_type_body_site, Treatment('sea water'))[T.fish GI]`
3. `C(sample_type_body_site, Treatment('sea water'))[T.fish digesta]`
4. `C(sample_type_body_site, Treatment('sea water'))[T.fish gill]`
5. `C(sample_type_body_site, Treatment('sea water'))[T.fish pyloric caeca]`
6. `C(sample_type_body_site, Treatment('sea water'))[T.fish skin]`

The last five fields of differentials (2–6) describe the association of features with samples from each of the studied body sites, using seawater samples as a reference via Treatment coding.

(For reference, the fourth differential field, `C(sample_type_body_site, Treatment('sea water'))[T.fish gill]`, is what is shown in the rank plot sub-figures in the main text.)

The first field, `Intercept`, is less easily interpretable and not particularly relevant to the case study; this field is produced automatically by Patsy (**https://patsy.readthedocs.io**), the library used by Songbird to represent input formulae as design matrices.

Songbird Mathematical Details

As is also described in (Morton *et al.*, 2019), the multinomial regression used in Songbird is given as follows:

$$\beta \sim N(0,\sigma)$$
$$\eta_i = \text{alr}^{-1}(X_i\beta)$$
$$Y_i \sim \text{Multinomial}(\eta_i)$$

where $\beta \in \mathbb{R}^{k\times d-1}$ represents the regression coefficients for d features and k covariates, X_i are the covariate measurements for each sample i, and Y_i are the feature counts in sample i. A normal prior with variance σ is used to regularize the regression coefficients. A maximum likelihood procedure is employed to identify the optimal regression coefficients.

The vectors $\beta_k \in \mathbb{R}^{d-1}$ are in alr coordinates, and as a result can be represented as compositions by $\text{alr}^{-1}(\beta_k) \in S^d$. In (Morton *et al.*, 2019), these vectors are referred to as *differentials*. Since ranking is shift invariant, the ordering of this composition is agnostic to the choice of reference frame. As a result, the features can be sorted by their coefficients in β_k. By default, these differentials are represented in clr coordinates.

The regression implemented in Songbird is similar to the methodology in other differential abundance tools, such as ALDEx2 (Fernandes *et al.*, 2014) and DESeq2 (Love *et al.*, 2014). The estimated regression coefficients from any of these tools can be visualized in Qurro: a fully worked example demonstrating the use of Qurro with ALDEx2 outputs is linked to from Qurro's Tutorials section of its README, located at **https://github.com/biocore/qurro**.

*To whom correspondence should be addressed. Email: robknight@ucsd.edu

© 2019 The Author(s)
This is an Open Access article distributed under the terms of the Creative Commons Attribution Non-Commercial License (http://creativecommons.org/licenses/by-nc/2.0/uk/) which permits unrestricted non-commercial use, distribution, and reproduction in any medium, provided the original work is properly cited.

Differential names

Due to some current technical limitations, Qurro (as of writing) changes or removes certain special characters like `[` or `'` from field names. This is why the gill differential field name shown in Qurro—`C(sample_type_body_site, Treatment(sea water))(T:fish gill)`—has a slightly different name than it did in Songbird's output. (This behavior is documented in Qurro's README, which is distributed with its source code at **https://github.com/biocore/qurro**.)

2. QURRO LOG-RATIO-SELECTION CONTROLS USED

The log-ratios selected in Figs. 1 and 2 were selected using Qurro's filtering controls in the following way.

Fig. 1 (*Shewanella* to *Synechococcales*)

1. The numerator was selected by filtering to features where the `Taxon` field contained the text `Shewanella`.
2. The denominator was selected by filtering to features where the `Taxon` field contained the text `Synechococcales`.

Fig. 2 (*Shewanella* to bottom $\sim 10\%$ features)

1. The numerator was selected by filtering to features where the `Taxon` field contained the text `Shewanella`.
2. The denominator was selected by filtering to features where the gill differential value—that is, `C(sample_type_body_site, Treatment(sea water))(T:fish gill)`—was less than `-2.102`. (This value was chosen in order to make the denominator include exactly the bottom 98 features.)

Screenshots of using these controls in Qurro are shown in Supplementary Figs. 1 and 2.

Screenshot Details

As the warning shown on the left side of the screenshots in Supplemental Figs. 1 and 2 explains, the rank plot in these screenshots has been scaled so that each bar (feature) has a width of less than 1 pixel: this is done in order to show more of the rank plot on the screen at once. Unchecking the `Fit bar widths to a constant plot width?` checkbox resets the bar widths in the rank plot to a larger value comparable to that shown in Figs. 1(a) and 2(a), albeit one that results in the full rank plot not being visible all at once on most screens without horizontally scrolling.

These visualizations were generated using Qurro version 0.6.0 and are displayed here on a macOS 10.15.3 laptop using Google Chrome version 80.0.3987.132. When taking these screenshots, the browser was zoomed out somewhat to show more of the controls and the `Numerator Features` table at the bottom-left of the screen was scrolled to the right to show selected numerator features' classified taxonomy information.

3. DETAILS ON QURRO (AND SONGBIRD) INPUT DATA FILTERING

Running Qurro requires a few distinct input files (or QIIME 2 artifacts, if running it as a QIIME 2 plugin): a feature table, a "rankings" file, a sample metadata file, and optionally a feature metadata file.

If any features within the feature table are not present in the input rankings, then Qurro will not include these features in the output visualization (since they would not be displayable on the rank plot). This means that, although Qurro doesn't impose very strict filtering guidelines on its own by default, the filtering behaviors of upstream "ranking" tools will necessarily impact the amount of data shown in Qurro.

Since this impacts the case study, we go into detail about this behavior here.

Songbird's `--min-feature-count`

For the case study dataset described in the manuscript, there were 23,253 features present in the feature table before running Songbird. However, Songbird applies a default `--min-feature-count` (i.e. the minimum number of samples a feature must appear in) of 10: this resulted in a large amount of features being removed from the visualization due to only appearing in a handful of samples. This is why there are just 985 features in the resulting Qurro visualization. (When generating a Qurro visualization, Qurro will output details explaining—if applicable—why certain samples/features have been removed from the visualization.)

Why aren't there any seawater samples shown in the paper figures?

One of the things we noticed midway through this case study was that *Shewanella* spp., for the most part, did not appear in seawater samples. To help explain this, we prepared a Jupyter Notebook (Kluyver *et al.*, 2016) that shows why these samples have been dropped. This notebook is available in the repository **https://github.com/knightlab-analyses/qurro-mackerel-analysis**.

Non-numeric `age_2` values. Since the `age_2` field refers to the estimated age of a sample's host fish, this field is not meaningful for non-fish samples like seawater. As shown in the notebook, all of the 50 seawater samples in our feature table have a non-numeric `age_2` value—this is one of the "reasons" Qurro has for dropping samples from the sample plot, and it explains why seawater samples cannot be shown in Figs. 1(c) or 2(c).

Relative lack of Shewanella features. As shown in the notebook, only one of the 50 seawater samples in our feature table included a feature classified as *Shewanella*. This particular *Shewanella* feature only appears in two samples in the feature table (including the aforementioned seawater sample), so it is not ranked by Songbird due to the default `--min-feature-count` described above.

From the Qurro visualization's perspective, then, none of the seawater samples contains any *Shewanella* features—so visualizing both of the log-ratios shown in the paper's case study will necessarily involve filtering out all of the seawater

samples, unless imputation of some form were to be used. This is the reason why seawater samples are not shown in Figs. 1(b) and 2(b), and it's a reason (in addition to the `age_2` reason) why seawater samples are not shown in Figs. 1(c) and 2(c).

Reflection on this phenomenon. We note that the large amount of samples dropped here was likely caused in part by the nature of the case study. Since in general different body sites are expected to harbor different microbial communities, it makes sense that taxa common in one sample type might go almost or completely undetected in other sample types.

When looking for differentially abundant taxa across more subtly different sample categories (e.g. skin samples at different timepoints in the progression of atopic dermatitis, as shown in (Morton *et al.*, 2019)), we expect that sample dropout like what we observed with seawater samples here will be less of an issue.

REFERENCES

Fernandes, A. D., Reid, J. N., Macklaim, J. M., McMurrough, T. A., Edgell, D. R., and Gloor, G. B. (2014). Unifying the analysis of high-throughput sequencing datasets: characterizing RNA-seq, 16s rRNA gene sequencing and selective growth experiments by compositional data analysis. *Microbiome*, **2**(1), 15.

Kluyver, T., Ragan-Kelley, B., Pérez, F., Granger, B. E., Bussonnier, M., Frederic, J., Kelley, K., Hamrick, J. B., Grout, J., Corlay, S., and others (2016). Jupyter Notebooks-a publishing format for reproducible computational workflows. In *ELPUB*, pages 87–90.

Love, M. I., Huber, W., and Anders, S. (2014). Moderated estimation of fold change and dispersion for rna-seq data with deseq2. *Genome biology*, **15**(12), 550.

Morton, J. T., Marotz, C., Washburne, A., Silverman, J., Zaramela, L. S., Edlund, A., Zengler, K., and Knight, R. (2019). Establishing microbial composition measurement standards with reference frames. *Nature Communications*, **10**(1), 2719.

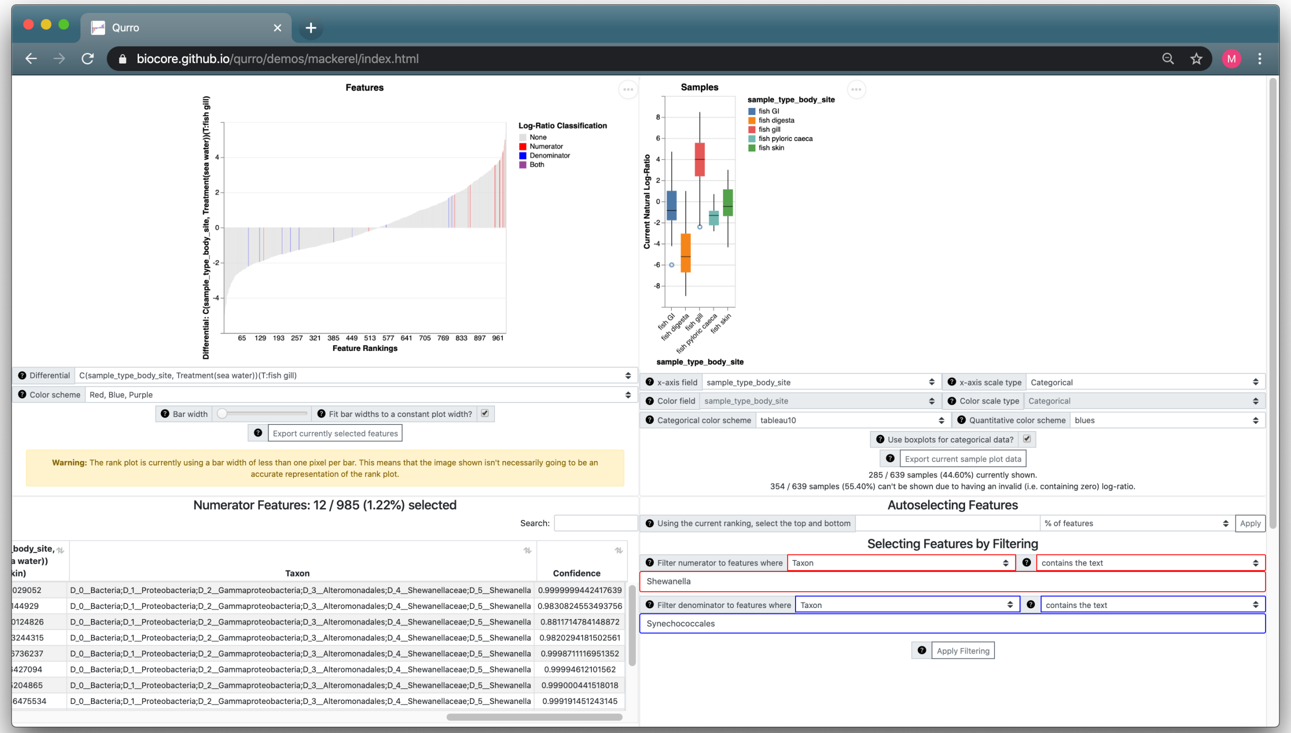

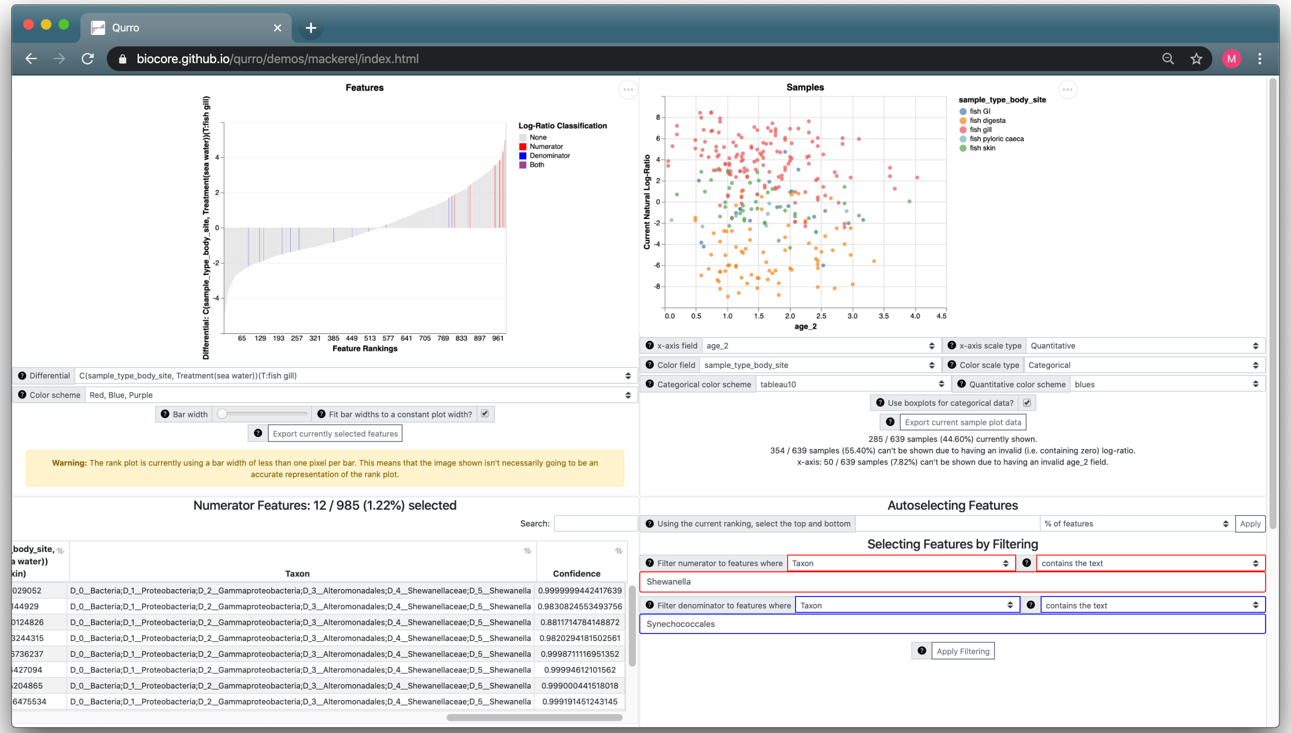

Supplementary Figure 1. Screenshots of a Qurro visualization of the case study data, showing the controls used to recreate Figs. 1(a–c) from the main text. Note the text entered in the `Selecting Features by Filtering` section at the bottom-right of the screen, which shows the textual queries used to select a log-ratio of *Shewanella* features to *Synechococcales* features.

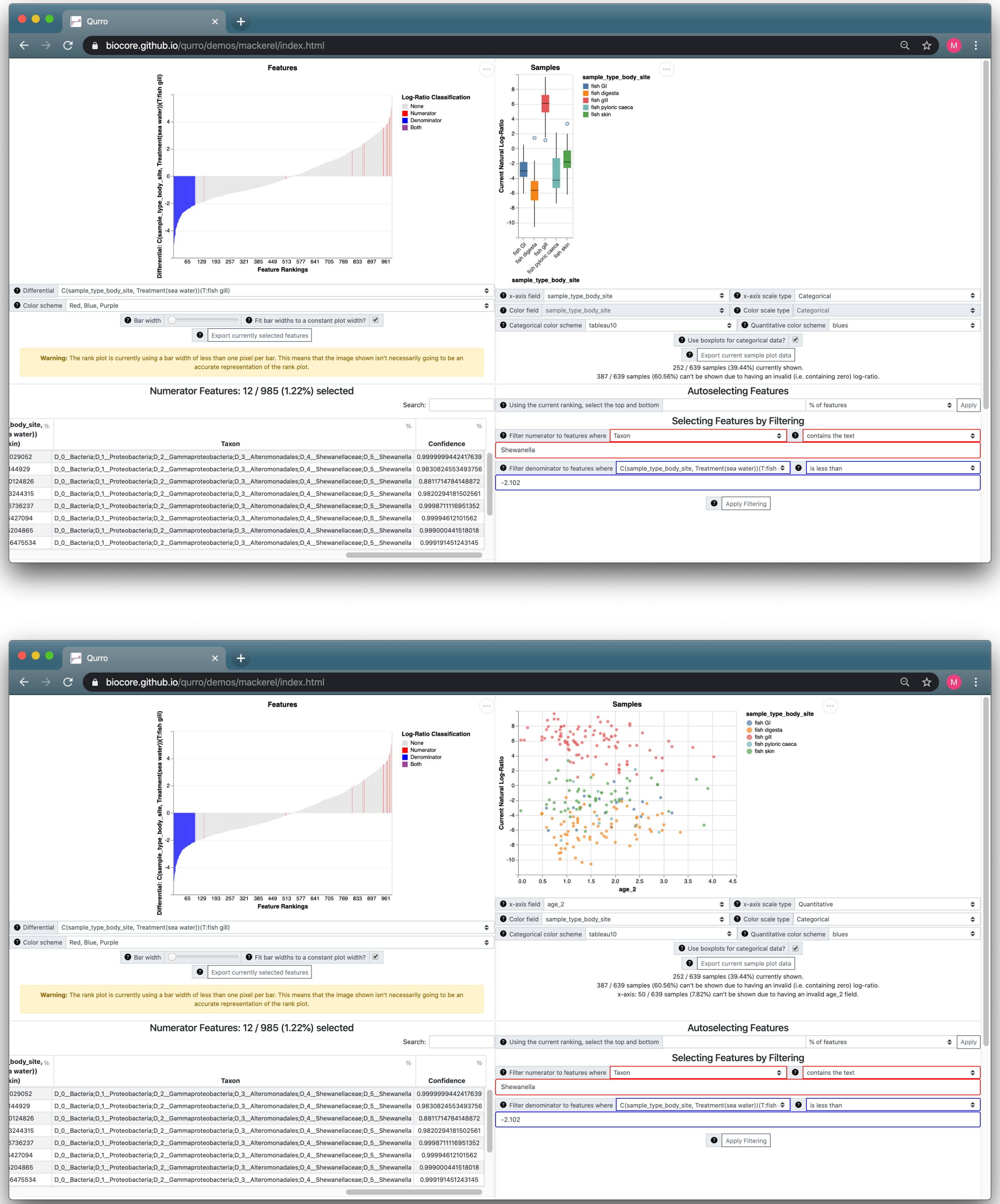

Supplementary Figure 2. Screenshots of a Qurro visualization of the case study data, taken analogously to those in Supplementary Fig. 1 but this time showing the controls used to recreate Figs. 2(a–c) from the main text. The difference between these screenshots and those in Supplementary Fig. 1 is due to the selected denominator features, which now comprise the bottom 98-ranked features for the gill differentials rather than the classified *Synechococcales* features. Although it is cut off somewhat by the dropdown's width, the first dropdown after the `Filter denominator to features where` label indicates that the `C(sample_type_body_site, Treatment(sea water))(T:fish gill)` differential field is selected.
